# The Age of Uterine Disease

**Published:** 1861-12

**Authors:** 


### THE AGE OF UTERINE DISEASE.

It has been remarked by a popular writer that this is "the age of uterine disease." In the medical profession, and with the other sex, the assertion certainly is not wide of the truth.

Uterine diseases have been the all-engrossing theme of a large class of practitioners for many years. Volumes have been written upon these affections, with chaste or unchaste illustrations of every grade, from the secret and undetermined forms of sterility, to the gravest forms of cancer; interminable discussions have been held upon the ever-varying phases of the diseases of this organ; and students of uterine pathology have always been rewarded with rich discoveries in fecund placer. If we were to believe all that is written of the inherent and acquired diseases of the organ, on the integrity of which depends the perpetuation of our species, how surely fated to early extinction would seem the human race? If it be perpetuated, it would be through decaying germs that must give origin to imperfect forms and decrepid generations.

But while it is true that uterine diseases exist and form a large class of affections which are capable of destroying the health and happiness of the sex, can any observant practitioner doubt that the uterus is, in our time, the scapegoat of many a latent malady of the female that is not correctly diagnosticated? Said an eminent obstetrician of this city: "If I should confirm the diagnosis in every case that is sent to me from the country, as one of undoubted uterine disease, I could add thousands of dollars to my annual income." He was emphatic in the expression of his opinion, that medical men, nowadays, conveniently referred to the womb a vast number of affections of which they either had not the tact or knowledge to determine the seat and nature. He examined the consulting patient with an habitual anticipation of finding a normal condition. Such statements are startling, and indicate a vast amount of carelessness or ignorance, or both, in the medical profession. In general, no diseases are more readily susceptible of accurate diagnosis than those peculiar to the uterus. They belong, in fact, to the diseases distinguished by the French as External Pathology. If there is an ulcer on the parts it is seen as distinctly as if on the leg; if there is unnatural enlargement, it is as detectible as a swollen finger; if there is a tumor of any kind or description, it is as demonstrable as a similar growth on the face; if there is displacement in any direction, it as as apparent as a dislocated limb. Indeed, a physician, with all the mechanical aids which we now possess for investigating uterine diseases, cannot be held guiltless of culpable ignorance who pronounces falsely upon the presence of grave lesions. He has no excuse for diagnosticating an ulcer when there is none; or prolapsus, when the organ is in a normal position; os anteflexion or retroflexion, when neither exists. And yet these false opinions are, it must be admitted, daily given, greatly to the discredit of many a physician in the eyes of an honest and competent expert. We believe that these errors are generally the result of carelessness. There is in many, also, a disposition to give always a definite opinion, especially in an obscure case; and it is convenient to fix upon an organ which has the popular acknowledgment of being the happy abode of all the undiscovered maladies of the female organization. The uterus has now come to enjoy the relative position of the liver in its ability of concentrating within itself all the undefinable diseases to which the sex are subject. Although the term "liver complaint" has now become obsolete in the nomenclature of many practitioners, yet its place is more than supplied by the phrase "uterine disease."

Aside from the humiliation of professional character which results from such ignorance and carelessness, there are other evils of a very different kind that must not be overlooked. We have thereby opened a large and fertile field for the special advantage of quackery in its lowest and most revolting forms. It is not strange that the interesting and interested subjects of the affections have become alarmed at the almost universal prevalence of the belief in the disabilities peculiar to their unfortunate sex. Thousands of nervous ladies suffering from some slight and obscure derangement of digestion, or other departure from health, are secretly informed by friends that the womb, that mysterious organ, with its innumerable susceptibilities, is liable to an infinite number of strange disorders. At once a mania for an investigation seizes the individual victim, which nothing but the manipulations with the speculum can relieve. And alas! too often instead of relieving a proper apprehension on the part of the patient, even though she is correctly informed that the womb is not diseased, a new source of excitement is established which is far more dangerous to her happiness than actual disease. If her aliments are lightly treated by her medical attendant she readily falls into the hands of a vulgar irregular, and becomes the dupe of his villanous machinations. In more than one instance has the profession of this city witnessed a uterine furor, created by an unblushing quack, which neither reason nor modesty could control. And but recently we noticed an instance in which a most ignorant pretender opened a hospital for the treatment of uterine tumors, in one of the most intelligent and moral communities of an interior state; crowds of women flocked to him, and all were found to be suffering from tumors of the womb. By accident a patient more intelligent than others, discovered that the tumor was a piece of raw meat, which was introduced at the first examination, and which, after long treatment, was removed to the great relief of the patient.

It is time that uterine pathology was thoroughly understood by every practitioner. It is not, as we have already intimated, difficult to learn so thoroughly that mistakes in diagnosis will be only exceptions, and not, as now, the rule.—*American Medical Times.*

---

Amount of Army Rations per Month. — The following figures show the amount of commissary stores which will be consumed in one month by the United States army when brought up to the standard authorized by Congress, viz. 500,000 men. It will be seen that the labors of the commissary department are anything but trivial, and that the cost of feeding an army is a somewhat serious item. 11,250,000 pounds of pork, or 18,750,000 pounds of fresh beef; 105,380 barrels of flour; 37,500 bushels of beans, or 1,500,000 pounds of rice; 1,500,000 pounds of coffee; 2,250,000 pounds of sugar; 150,000 gallons of vinegar; 225,000 pounds of candles; 600,000 pounds of soap; 6,384 bushels of salt, and 6,600,000 pounds of potatoes.

---

*Books for Sale.*—Any one wishing to procure a full set of the "American Journal of Medicine and Collateral Sciences," Edited by I. Hays, M. D., Philadelphia, from Jan. 1st, 1847, to the close of the present volume, 1861, well bound and in perfect preservation; or the "London Lancet," American reprint, from Jan., 1847, to 1855; or the "Boston Medical Journal," '47 to '57; or the first twelve parts of "Braithwaite's Retrospect," all well bound and in good order, can procure the same, at low figures, by application to the Editors of this Journal, or addressing a line to box 4458 Chicago P. O.

